# Effectiveness of extracorporeal shock wave therapy for temporomandibular disorders: a systematic review and meta-analysis

**DOI:** 10.22514/jofph.2026.036

**Published:** 2026-05-12

**Authors:** Shunyuan Liu, Yuli Shuai, Liu Ye, Shiqi Tan, Yizhan Wang, Dandong Wu

**Affiliations:** ^1^The First Affiliated Hospital of Chongqing Medical University, 400016 Chongqing, China; ^2^Department of Rehabilitation Medicine, Key Laboratory of Physical Medicine and Precision Rehabilitation of Chongqing Municipal Health Commission, The First Affiliated Hospital of Chongqing Medical University, 400016 Chongqing, China; ^3^Department of Rehabilitation Medicine, The First Affiliated Hospital of Chongqing Medical University, 400016 Chongqing, Chin; ^4^CQMU—University of Leicester Joint Institute, Chongqing Medical University, 400016 Chongqing, China

**Keywords:** Extracorporeal shock wave therapy, Temporomandibular disorders, Systematic review, Meta-analysis

## Abstract

**Background**: Extracorporeal shock wave therapy (ESWT) is commonly employed for treating various musculoskeletal disorders; however, its effectiveness in managing temporomandibular disorders (TMD) remains inconclusive. This study aimed to assess the effectiveness of ESWT for TMD by reviewing randomized clinical trials. **Methods**: Systematic searches were conducted across seven databases from inception to April 2025 to identify randomized clinical trials (RCTs) evaluating ESWT in patients with TMD. Eligible studies were required to report at least one of the following outcomes: visual analog scale (VAS) for pain or maximum mouth opening (MMO). A meta-analysis was performed, categorizing results by treatment type (ESWT alone *vs.* ESWT combined with other treatments). The risk of bias and study quality were assessed using Review Manager version 5.4.1, and all analyses were conducted using R v.4.4.3 (R Project for Statistical Computing). **Results**: Fourteen RCTs (n = 1107 participants) were included. Compared to active controls, ESWT did not result in significant improvements (VAS: mean difference (MD) = −0.81; 95% confidence interval (CI) = −1.77 to 0.16; *p* = 0.06, *I*^2^ = 0.0%; MMO: Standardized Mean Difference (SMD) = −1.84; 95% CI = −18.43 to 14.74; *p* = 0.39, *I*^2^ = 97.5%). However, when used as an adjunctive therapy, ESWT significantly improved pain intensity and mouth opening (VAS: MD = 0.94; 95% CI = 0.61 to 1.26; *p* < 0.0001, *I*^2^ = 19.5%; MMO: SMD = 0.69; 95% CI = 0.48 to 0.90; *p *< 0.0001, *I*^2^ = 45.5%). **Conclusions**: ESWT, when used as an adjunctive therapy for TMD, significantly alleviates pain and improves mouth opening. However, these results should be interpreted with caution due to the generally low quality of the included studies and the absence of long-term follow-up data. This meta-analysis synthesizes the available evidence, highlights critical research gaps, and provides cautious recommendations for clinical practice. **The PROSPERO Registration**: CRD42025631405.

## 1. Introduction

Temporomandibular disorders (TMD) refer to a group of musculoskeletal disorders affecting the temporomandibular joint (TMJ), masticatory muscles, and surrounding anatomical structures [1, 2]. Patients commonly present with clinical symptoms such as irregular jaw movement, facial pain, restricted mouth opening, joint noise, and headaches. These manifestations significantly impact quality of life, particularly due to pain during chewing [3, 4]. The etiology of TMD is multifactorial and sometimes controversial, involving traumatic injuries, abnormal muscle hyperactivity, intra-articular disc disorders, systemic diseases, hormonal influences, and psychological factors [5, 6, 7]. Alrizqi AH *et al*. [8] reported that 30%–50% of the global population may be affected by TMD, with a higher prevalence in women, and that its onset may be associated with psychological status. The condition typically affects individuals aged 20 to 50, although it can also occur in adolescents [3].

Management of TMD involves a range of approaches, with primary objectives including pain relief, improved mouth opening, and enhanced quality of life for patients [9]. Treatment options encompass jaw physiotherapy, medications, patient education and self-care, occlusal appliance therapy, behavioral therapy, psychotherapy, minimally invasive procedures (*e.g.*, arthrocentesis and intra-articular injections), and TMJ surgery [10, 11]. Despite the variety of treatments, they all have certain limitations. For instance, nonsteroidal anti-inflammatory drugs (NSAIDs) may cause gastrointestinal issues and negatively affect liver and kidney function, while occlusal splint therapy is contraindicated in patients with obstructive sleep apnea. Minimally invasive interventions, such as intra-articular injections, carry risks of local infection or tissue irritation [11], and surgical options are costly, invasive, and involve prolonged recovery times [12].

Extracorporeal Shock Wave Therapy (ESWT) is a non-invasive physical therapy technique that uses high-energy shock waves to target specific tissues, promoting healing, alleviating pain, and improving function. Initially developed for the treatment of kidney stone in the 1980s [13], ESWT was serendipitously found to induce an osteogenic response in animal models, which led to its application in treating musculoskeletal disorders [14, 15]. Although the precise therapeutic mechanism of ESWT remains unclear, its effectiveness in treating musculoskeletal conditions, such as proximal plantar fasciitis, lateral epicondylitis of the elbow, calcifying tendinitis of the shoulder, patellar tendinopathy, and Achilles tendinopathy, is well-established [14, 15].

As early as 2002, ESWT was explored for the treatment of TMJ locking [16, 17]. In this context, the ESWT probe, coupled with gel, is applied to TMJ tender points or adjacent masticatory muscles, while avoiding the facial nerve to minimize risks, in accordance with clinical guidelines [18]. A typical session lasts 5–10 minutes at a moderate energy level, enabling shockwaves to reach the TMJ synovium and articular cartilage [19]. Reported side effects are mild and transient, such as local erythema, slight pain, and edema, with no severe complications, such as nerve injury or joint damage, reported to date [18]. Recent studies have demonstrated that ESWT may offer a protective effect on subchondral bone structure and cartilage in rat models of TMJ osteoarthritis [20]. Clinical studies comparing ESWT with other treatment options for TMD are also ongoing.

To date, no systematic reviews or meta-analyses have been conducted on this topic. This study aims to evaluate the efficacy of ESWT in the treatment of TMD based on available RCTs, following the Preferred Reporting Items for Systematic Reviews and Meta-Analyses (PRISMA) guidelines.

## 2. Materials and methods

This systematic review and meta-analysis was registered in PROSPERO (International Prospective Register of Systematic Reviews, CRD42025631405) and conducted in accordance with PRISMA guidelines (**Supplementary material 1**).

### 2.1 Study eligibility criteria

Inclusion criteria were as follows: (1) Randomized controlled trials (RCTs); (2) Diagnosis of TMD confirmed by either validated diagnostic criteria (*e.g.*, Diagnostic Criteria for Temporomandibular Disorders (DC/TMD), Research Diagnostic Criteria for Temporomandibular Disorders (RDC/TMD)) or clear clinical description of TMD-related signs and symptoms (*e.g.*, pain in the temporomandibular joint area, jaw dysfunction); (3) Any study group receiving ESWT; (4) Adult participants (≥18 years); (5) Outcome measures including at least one of the following: visual analog scale (VAS) or maximum mouth opening (MMO). Exclusion criteria were as follows: (1) History of surgical treatment for TMD; (2) Presence of other major medical conditions (*e.g.*, cerebral infarction, myocardial infarction, heart failure); (3) full-text articles that were unavailable.

### 2.2 Data sources and search strategy

Systematic searches were performed across seven electronic databases: PubMed (inception (1968)—01 April 2025), Embase (inception (1988)—01 April 2025), Cochrane Central Register of Controlled Trials (CENTRAL, inception (1991)—01 April 2025), China National Knowledge Infrastructure (CNKI, inception (1999)—01 April 2025), VIP Chinese Science and Technology Periodical Database (VIP, inception (2000)—01 April 2025), Chinese Biomedical Literature Database (SinoMed, inception (1978)—01 April 2025), and Wanfang Data (inception (1988)—01 April 2025).

No language restrictions were applied. Keywords and Medical Subject Headings (MeSH) were identified using the PubMed MeSH Database. The core search employed a “MeSH term + free-text word” strategy, structured into three focused modules for accuracy and comprehensiveness: (1) Disease: TMD and its synonyms/diagnostic terms; (2) Intervention: only ESWT, including its full name, abbreviation, and synonyms; (3) Study type: RCTs exclusively, filtered via database tools and keywords to exclude non-RCTs. A detailed search strategy is provided in **Supplementary material 2**.

### 2.3 Study selection and data extraction

All retrieved articles were imported into reference management software (EndNote 20.6, Build 17174, Clarivate, Philadelphia, PA, USA). Two independent investigators (LSY and SYL) removed duplicates and screened titles and abstracts against the eligibility criteria. They then independently reviewed the full texts to select eligible studies.

Upon identifying eligible articles, the two independent reviewers (LSY and SYL) assessed the risk of bias using the Cochrane Risk of Bias Tool (implemented in Review Manager 5.4). Data from eligible studies were extracted into a pre-designed Excel spreadsheet, including: study authors, publication year, sample characteristics (sample size, gender distribution, and age), diagnostic criteria, intervention details, and primary and secondary outcomes. Any discrepancies were resolved through discussion between the two investigators. If agreement could not be reached, a third author (YL) participated to achieve consensus.

### 2.4 Data analysis

The quality of the included studies was assessed using the Cochrane Collaboration’s Risk of Bias Assessment Tool for Randomized Trials, implemented in RevMan 5.4 (Nordic Cochrane Centre, Cochrane Collaboration). All statistical analyses were conducted using R software v.4.4.3 (R Project for Statistical Computing) with the meta package. The primary outcome was pain intensity, measured by the VAS, and the secondary outcome was MMO. Effect sizes were reported as mean differences (MD) or standardized mean differences (SMD) with corresponding 95% confidence intervals (CIs), with a two-tailed *p*-value of < 0.05 considered statistically significant. Forest plots were used to visually present study results for intuitive comparison and interpretation.

Heterogeneity was evaluated using Cochrane’s *Q* test and the *I*^2^ statistic. Sensitivity analysis was performed using the leave-one-out approach (excluding one study at a time and re-computing the pooled effect size) to assess the robustness and stability of the meta-analysis results. Publication bias was examined visually using funnel plots and quantitatively verified by Egger’s linear regression test, with a *p*-value > 0.05 indicating no significant publication bias.

To assess the certainty of evidence for the primary and secondary outcomes, the GRADE (Grading of Recommendations Assessment, Development and Evaluation) approach was employed. Evidence quality was classified as high, moderate, low, or very low based on key domains including risk of bias, heterogeneity, indirectness, imprecision, and publication bias. These judgments were succinctly summarized alongside the meta-analysis results.

## 3. Results

### 3.1 Study selection

A total of 64 potentially relevant records were retrieved from the databases. After removing duplicates, 29 records remained. Nine records were excluded after title and abstract screening due to irrelevant interventions or incomplete outcome data, leaving 20 full texts for further eligibility assessment. Of these, 6 were excluded based on predefined eligibility criteria: 3 had incomplete outcome data, and 3 reported inadequate interventions. Ultimately, 14 studies were included in the meta-analysis [21, 22, 23, 24, 25, 26, 27, 28, 29, 30, 31, 32, 33, 34]. The study selection process is outlined in Fig. 1.

**Fig. 1.** PRISMA flow diagram. The PRISMA flow diagram illustrates the study selection process. A total of 64 articles were retrieved from the bibliographic search, and 14 articles were included in the review. PRISMA: Preferred Reporting Items for Systematic Reviews and Meta-Analysis; PubMed: US National Library of Medicine; Embase: Excerpta Medica Database; CNKI: China National Knowledge Infrastructure; VIP: Chinese Science and Technology Periodical Database; SinoMed: Chinese Biomedical Literature Database; Wanfang: Wanfang Data Knowledge Service Platform.

### 3.2 Characteristics of included studies

The 14 included studies [21, 22, 23, 24, 25, 26, 27, 28, 29, 30, 31, 32, 33, 34] were all RCTs published between 2016 and 2024, comprising a total of 1107 patients with a balanced gender distribution. All participants were diagnosed using diagnostic criteria such as the DC/TMD (with subtypes I, II, I + II, or unspecified) and the RDC/TMD (subtypes I, III, or unspecified), with one study using the specific description “temporomandibular joint pain with mandibular dysfunction”. Interventions primarily focused on ESWT (either alone, combined with movement therapy, or paired with additional treatments like NSAIDs, antispasmodics, splints, electroacupuncture, or TENS (Transcutaneous Electrical Nerve Stimulation)), with a treatment duration of 2–4 weeks. Outcomes were measured using 14 indicators (*e.g.*, VAS, MMO, FI (Friction Index)), with each study selecting 2–6 indicators. All participants in the intervention group received ESWT, and 9 studies incorporated manual therapy as part of the treatment strategy. The main characteristics of the included studies are summarized in Table 1.

**Table 1.** Demographic characteristics of included studies.

First Author and Year	Participants Group	Sample of Male/Female	Mean (±SD) age (in years)	Diagnosis	Treatments	Treatment Duration (Weeks)	Study’s outcomes
Serap Keskin Tunç, 2024							
	ESWT	8/17	27.0 (±11.0)	DC/TMD I + II	ESWT + CG	4	A,B
	CG	5/31	23.9 (±7.1)		NSAID +Antispasmodic +a stabilization splint		
Peijie Han, 2018							
	ESWT	16/4	35.7 (±12.2)	DC/TMD I	ESWT + CG	2	A,B,C
	CG	7/13	33.3 (±11.3)		Electroacupuncture treatment		
Yuhang Chen, 2022							
	ESWT	26/25	37.8 (±4.0)	DC/TMD I	ESWT + CG	4	A,C,D
	CG	27/24	39.0 (±4.0)		Modality therapy +Manual therapy +Movement therapy		
Wei Song, 2024							
	ESWT	70/50	39.4 (±4.3)	DC/TMD I/II	ESWT + CG	2	A,B,C,E,F
	CG	68/52	39.3 (±4.1)		Modality therapy +Manual therapy +Movement therapy		
Bin Xu, 2016							
	ESWT	32/28	46.3 (±4.1)	Pain in the temporomandibular joint area. Mandibular dysfunction.	ESWT + CG	4	A,C,H,I,J
	CG	34/26	46.1 (±4.0)		Ultrashort wave therapy +Manual therapy		
Pu Wang, 2020							
	ESWT	9/11	45.2 (±6.9)	DC/TMD I	ESWT + CG	3	A,H,C
	CG	12/8	44.3 (±8.6)		Transcutaneous electrical nerve stimulation treatment + Manual therapy		
Pengcheng Wang, 2024							
	ESWT	10/33	37.8 (±10.6)	DC/TMD	ESWT + CG	4	A,B,C,F,G
	CG	14/29	36.8 (±9.9)		Glucosamine Hydrochloride Tablets +Ultrasound therapy +Manual therapy		
Wenjie Su, 2022							
	ESWT	12/18	28.4 (±6.8)	DC/TMD I + II	ESWT + CG	4	A,B,L,E
	CG	10/20	28.2 (±7.1)		Ultrashort wave therapy +Manual therapy +Ultrasound therapy		
Bin Hu, 2023							
	ESWT	12/8	29.9 (±1.8)	DC/TMD	ESWT + CG	3	A,B,C
	CG	13/7	30.0 (±1.9)		Movement therapy		
Bin Hu, 2023							
	ESWT	6/14	33.8 (±16.9)	DC/TMD	ESWT + CG	2	A,B,C
	CG	2/18	33.2 (±15.2)		Modality therapy +Manual therapy +Movement therapy		
Yuxia Zhu, 2021							
	ESWT	22/17	34.8 (±10.5)	RDC/TMD I	ESWT +Movement therapy	3	A,J
	CG	21/18	35.1 (±10.3)		Ultrashort wave therapy +Movement therapy		
Wenyan Li, 2019							
	ESWT	6/14	35.0 (±8.05)	RDC/TMD III	ESWT + Movement therapy	4	A,B,C
	CG	7/13	33.4 (±10.1)		Ultrashort wave therapy +Movement therapy		
Wenyan Li, 2020							
	ESWT	14/26	25.8 (±7.7)	RDC/TMD	ESWT	4	A,K
	CG	12/28	35.0 (±8.0)		Ultrashort wave therapy		
Lifei Liu, 2018							
	ESWT	31/49	27–50	RDC/TMD I	ESWT	2	A,B,C
	CG				Ultrashort wave therapy +Ultrasound therapy		

*ESWT: extracorporeal shock wave therapy; CG: control group; RDC/TMD: Research Diagnostic Criteria for Temporomandibular Disorders (I: Muscle Disorders; II: Disc Displacements; III: Other Common Joint Disorders); DC/TMD: Diagnostic Criteria for Temporomandibular Disorders (I: Painful Disorders; II: Joint Disorders; III: Other Disorders); NSAIDs: nonsteroidal anti-inflammatory drugs; A: Visual analog scale (VAS); B: Maximum mouth opening (MMO); C: Friction Index (FI); D: Helkimo Index; E: Mandibular Function Impairment Questionnaire (MFIQ); F: Craniomandibular index (CMI); G: Oral Health Impact Profile-14 (OHIP-14); H: Opening range(OR); I: Lateral movement; J: Medical Outcomes Study 36-Item Short-Form Health Survey (SF-36); K: Temporomandibular opening index; L: Pressure pain threshold; SD: Standard Deviation.*

### 3.3 Risk of bias assessment of included studies

The risk of bias of the included studies was assessed using the Cochrane Risk of Bias Tool, with the results presented in Fig. 2 and **Supplementary Fig. 1**. Most studies exhibited an unclear risk of bias across multiple domains. While all 14 studies employed randomization for participant allocation, none specified whether allocation concealment was implemented. Only 2 studies [21, 33] provided details on the blinding method, both being assessed as having a high risk of performance bias but a low risk of detection bias. Complete outcome data were reported in only 1 study [28], and the risk of attrition bias remained unclear for the remaining 13 studies. Two studies [21, 33] were deemed to have a low risk of reporting bias, with the risk of reporting bias remaining unclear for the others.

**Fig. 2.** Risk of bias summary. The judgments for each bias item from the risk of bias assessment tool were made for all included studies. +: low risk of bias; −: high risk of bias; ?: unclear risk of bias.

### 3.4 Meta-analysis result

#### 3.4.1 Treatment effect of ESWT alone

Of the 14 RCTs included in the qualitative synthesis, 2 were subjected to meta-analysis to assess the efficacy of ESWT alone. In these studies, the control groups received shortwave diathermy and/or ultrasound therapy [33, 34]. Given the small number of included studies (n = 2), sensitivity analysis and publication bias assessment were not performed, as these methodological evaluations lack statistical validity and meaningful interpretability with such a limited sample size. Therefore, the certainty of evidence for these outcomes is very low (**Supplementary Tables 1,2**), primarily due to the small sample size and insufficient data to assess key quality aspects. Considering the limited number of trials, this subgroup analysis should be regarded as exploratory, and the results must be interpreted with caution.

##### 3.4.1.1 Pain

Both RCTs used the VAS to measure pain changes before and after treatment. The meta-analysis results revealed no statistically significant difference in pain reduction between the ESWT group and the control group (MD = −0.81; 95% CI = −1.77–0.16; *p* = 0.06; *I*^2^ = 0.0%; Fig. 3).

**Fig. 3.** Forest plot of ESWT alone for pain. Pooled mean differences were calculated using a random effects model. CI: confidence interval; MD: mean difference; SD: standard deviation.

##### 3.4.1.2 Maximum mouth opening (MMO)

Both RCTs also used MMO as an outcome indicator to assess improvements in TMJ function. The meta-analysis results for MMO indicated that, compared to the control group, the ESWT group did not show a statistically significant improvement in MMO (SMD = −1.84; 95% CI = −18.43–14.74; *p* = 0.39; *I*^2^ = 97.5%; Fig. 4).

**Fig. 4.** Forest plot of ESWT alone for maximum mouth opening. Pooled mean differences were calculated using a random effects model. SMD: standardized mean difference; CI: confidence interval; SD: standard deviation.

#### 3.4.2 Effects of ESWT in conjunction with other treatments

Of the 14 RCTs included in the qualitative synthesis, 12 were analyzed to evaluate the effectiveness of ESWT combined with other therapies [21, 22, 23, 24, 25, 26, 27, 28, 29, 30, 31, 32]. Among these, 10 RCTs incorporated manual therapy as the adjunctive intervention, 1 combined ESWT with electroacupuncture [22], and 1 combined ESWT with NSAIDs, antispasmodic drugs, and splint therapy [21]. According to the GRADE assessment, the certainty of evidence for this subgroup was rated as low to very low (**Supplementary Tables 1,2**).

##### 3.4.2.1 Pain

All 12 RCTs used the VAS to compare changes in TMD-related pain between the control group and the combination treatment group. Meta-analysis results indicated that, compared to the control group, the combination treatment group (ESWT + other therapies) significantly alleviated pain, with low certainty of evidence (MD = 0.94; 95% CI = 0.61–1.26; *p* < 0.0001; *I*^2^ = 19.5%; Fig. 5). Leave-one-out sensitivity analysis confirmed the stability and robustness of the pooled results, as sequential exclusion of individual studies did not alter the core findings (**Supplementary Fig. 2**). Publication bias was negligible: the funnel plot (**Supplementary Fig. 3**) showed a roughly symmetric distribution of studies, and Egger’s linear regression test yielded *t* = 0.29, degrees of freedom (*df*) = 10, *p* = 0.7800, with a small bias estimate of 0.2739 (Standard Error (SE) = 0.9546).

**Fig. 5.** Forest plot of ESWT in conjunction with other treatments for pain. Pooled mean differences were calculated using a random effects model. CI: confidence interval; MD: mean difference; SD: standard deviation.

##### 3.4.2.2 Maximum mouth opening (MMO)

Ten of the 12 RCTs [21, 22, 23, 24, 25, 27, 28, 29, 30, 32] reported MMO as an outcome indicator to assess TMJ functional improvement. Meta-analysis results showed that the combination treatment group (ESWT + active therapy) significantly increased MMO compared to the control group, with very low certainty of evidence (SMD = 0.69; 95% CI = 0.48–0.90; *p* < 0.0001; *I*^2^ = 45.5%; Fig. 6). Leave-one-out sensitivity analysis (**Supplementary Fig. 4**) confirmed the robustness of these findings, as sequential exclusion of individual studies did not alter the direction or significance of the pooled SMD. For publication bias, the funnel plot (**Supplementary Fig. 5**) displayed a roughly symmetric distribution of studies, and Egger’s linear regression test (*t* = −1.26, *df* = 8, *p* = 0.2415) further suggested no significant publication bias (bias estimate = −1.7686, SE = 1.3982).

**Fig. 6.** Forest plot of ESWT in conjunction with other treatments for maximum mouth opening. Pooled mean differences were calculated using a random effects model. SMD: standardized mean difference; CI: confidence interval; SD: standard deviation.

## 4. Discussion

This is the first meta-analysis to systematically compare the efficacy of ESWT monotherapy and adjunctive therapy for TMD. Analysis of the included studies revealed that, compared to the control treatment group, the ESWT monotherapy group showed no statistically significant improvements in either VAS scores or MMO. In contrast, the combination treatment group (ESWT combined with other therapies) demonstrated statistically significant improvements in both VAS and MMO outcomes compared to the control group.

While most RCTs suggest that ESWT is effective for TMD, its precise mechanism of action remains unclear [35]. A study using an animal model to investigate the effect of ESWT on chondrocytes and TMJ osteoarthritis in rats found that ESWT downregulates the expression of pro-inflammatory factors [20, 36]. Another study identified three main pathways through which ESWT alleviates musculoskeletal disorders: first, by acting on nerve fibers to reduce pain; second, by stimulating angiogenesis to promote tissue healing; and third, by regulating calcium deposition [35, 37].

Notably, treatment parameters and device-dependent factors influence the efficacy of ESWT: energy flux density (EFD) determines the depth of penetration, with suboptimal levels (either too low or too high) potentially limiting efficacy or increasing tissue irritation; wavelength affects penetration efficiency, with shorter wavelengths facilitating deeper tissue reach [18]; treatment duration (5–10 minutes per session) balances energy accumulation and patient tolerance; and applicator design influences energy distribution. Focused-output applicators ensure concentrated delivery to targets, while design variations may contribute to heterogeneity in treatment efficacy [18]. As previously noted, ESWT delivered at a moderate clinical EFD can penetrate to a depth sufficient to reach the core anatomical structures of the human TMJ region. This is supported by *in vitro* evidence showing its ability to traverse soft tissues and superficial bone. This penetration depth aligns with the TMJ’s anatomical layers (skin, subcutaneous tissue, fascia, joint capsule, and articular disc), ensuring energy reaches key pathological targets to relieve pain, release intra-articular fibrosis, and reduce synovial inflammation [19].

Although ESWT can treat musculoskeletal diseases through multiple mechanisms, the results of this meta-analysis indicate that its therapeutic effect is not statistically significant when used alone. Several factors may explain this finding. First, the control interventions in the ESWT monotherapy subgroup were limited to ultrashort wave therapy and/or ultrasonic therapy. Ultrashort wave therapy promotes vasodilation, accelerates blood circulation, and improves the nutritional supply and metabolism of local tissues [38]. Ultrasonic therapy, through its mechanical and thermal effects, alleviates pain and promotes soft tissue regeneration [39]. The therapeutic effects of these control therapies overlap with those of ESWT, which may explain why ESWT alone did not show a statistically significant advantage over the control group in treating TMD. Furthermore, the studies included specifically enrolled patients with Type I TMD, whose primary symptom is pain. The lack of significant differences in treatment outcomes might therefore be attributed to the fact that ESWT, ultrasonic therapy, and ultrashort wave therapy are all established modalities for pain relief, potentially leading to similar effect profiles in this context.

In contrast, the meta-analysis revealed a statistically significant therapeutic effect when ESWT was combined with other treatments. Notably, 10 of the 12 studies in this subgroup employed manual therapy. Various manual therapy techniques, including TMJ mobilization, soft tissue manipulation, and massage of the masticatory and cervical muscles, are commonly used in TMD management. These techniques reduce connective tissue and muscle stiffness, thus improving functional capacity [40]. The addition of ESWT is believed to accelerate pain relief by modulating anti-inflammatory mediators and endorphins, enhancing patient compliance and tolerance for subsequent manual therapy [36]. Moreover, some patients with TMD experience involuntary contractions of the masseter and temporalis muscles during joint movement, which limits the therapist’s ability to improve joint range of motion [41]. By exerting anti-fibrotic effects and alleviating muscle spasms, ESWT promotes muscle relaxation, facilitating deeper and more targeted manual therapy [42, 43]. This synergistic effect has been observed in other musculoskeletal conditions, such as frozen shoulder [44], where ESWT, as an adjunct to conventional treatment, has shown statistically significant benefits in pain reduction and functional improvement.

This study has several limitations that must be considered when interpreting the results. First, the sample size of the ESWT monotherapy subgroup is severely limited, with only two studies (totaling 160 participants) meeting the inclusion criteria. As a result, the findings should be interpreted with caution. Second, all included studies were of overall low methodological quality, with an unclear risk of bias, primarily due to insufficient blinding of participants and researchers, as well as short follow-up durations. Third, substantial clinical heterogeneity existed across the included studies, including inconsistent TMD diagnostic subtypes and varying concomitant interventions, which may limit the generalizability of the pooled results. Fourth, heterogeneity in ESWT treatment parameters, including intensity, frequency, and total number of sessions, may have contributed to variations in therapeutic effects.

Despite these limitations, this meta-analysis provides clinicians with a structured synthesis of the available evidence on ESWT’s efficacy. The primary goal is not to establish definitive clinical recommendations but to clarify the current evidence profile and identify key research gaps. Rigorous methodological assessments were employed to ensure transparent reporting of limitations, enabling readers to accurately interpret the certainty of the findings. In light of these limitations, future research should prioritize well-designed, multicenter RCTs with large sample sizes, rigorous blinding, and long-term follow-up to definitively determine the therapeutic role of ESWT.

## 5. Conclusions

Compared to conventional treatments, ESWT monotherapy did not demonstrate a significant advantage in pain relief or improvement in mouth opening for patients with TMD; however, this finding is constrained by the low methodological quality and small sample size of existing studies. In contrast, ESWT as adjunctive therapy showed statistically significant benefits. Nevertheless, given the overall low certainty of available evidence, definitive conclusions regarding its efficacy cannot yet be established. Our results provide a structured synthesis of current evidence synthesis to inform clinical decision-making and highlight the need for future high-quality trials to establish conclusive evidence.

## Supplementary Material

Supplementary material associated with this article can be found, in the online version, at https://files.jofph.com/ files/article/2054075010740305920/attachment/ Supplementary%20material.zip.






