# Supplementary material for: Effectiveness of extracorporeal shock wave therapy for temporomandibular disorders: a systematic review and meta-analysis
Source: J Oral Facial Pain Headache. 2026 May 12;40(3):65–75. doi: 10.22514/jofph.2026.036 (PMC13223908; doi:10.22514/jofph.2026.036)
Supplement: Supplementary file 2 [file Supplementary-material-2.docx]

Supplementary material 2

# Database: PubMed (As of 16 November 2025).

| 8 | 3 AND 6 AND 7 | 2 |
| --- | --- | --- |
| 7 | randomized controlled trial[Publication Type] OR randomized[Title/Abstract] OR placebo | 1,137,021 |
| 6 | 4 OR 5 | 22,752 |
| 5 | ((((((((((((((((((((((Disorders, Temporomandibular Joint[Title/Abstract]) OR (Disorder, Temporomandibular Joint[Title/Abstract])) OR (Joint Disorders, Temporomandibular[Title/Abstract])) OR (Joint Disorder, Temporomandibular[Title/Abstract])) OR (Temporomandibular Joint Disorder[Title/Abstract])) OR (Temporomandibular Joint Diseases[Title/Abstract])) OR (Diseases, Temporomandibular Joint[Title/Abstract])) OR (Disease, Temporomandibular Joint[Title/Abstract])) OR (Joint Diseases, Temporomandibular[Title/Abstract])) OR (Joint Disease, Temporomandibular[Title/Abstract])) OR (Temporomandibular Joint Disease[Title/Abstract])) OR (TMJ Diseases[Title/Abstract])) OR (Diseases, TMJ[Title/Abstract])) OR (Disease, TMJ[Title/Abstract])) OR (TMJ Disease[Title/Abstract])) OR (Temporomandibular Disorders[Title/Abstract])) OR (Disorders, Temporomandibular[Title/Abstract])) OR (Disorder, Temporomandibular[Title/Abstract])) OR (Temporomandibular Disorder[Title/Abstract])) OR (TMJ Disorders[Title/Abstract])) OR (Disorders, TMJ[Title/Abstract])) OR (Disorder, TMJ[Title/Abstract])) OR (TMJ Disorder[Title/Abstract]) | 9152 |
| 4 | “Temporomandibular Joint Disorders”[Mesh] | 20,115 |
| 3 | 1 OR 2 | 3085 |
| 2 | ((((((((((((((Extracorporeal Shockwave Therapies[Title/Abstract]) OR (Shockwave Therapies, Extracorporeal[Title/Abstract])) OR (Shockwave Therapy, Extracorporeal[Title/Abstract])) OR (Therapy, Extracorporeal Shockwave[Title/Abstract])) OR (Extracorporeal Shock Wave Therapy[Title/Abstract])) OR (Shock Wave Therapy[Title/Abstract])) OR (Shock Wave Therapies[Title/Abstract])) OR (Therapy, Shock Wave[Title/Abstract])) OR (Extracorporeal High-Intensity Focused Ultrasound Therapy[Title/Abstract])) OR (Extracorporeal High Intensity Focused Ultrasound Therapy[Title/Abstract])) OR (High-Intensity Focused Ultrasound Therapy[Title/Abstract])) OR (High Intensity Focused Ultrasound Therapy[Title/Abstract])) OR (HIFU Therapy[Title/Abstract])) OR (HIFU Therapies[Title/Abstract])) OR (Therapy, HIFU[Title/Abstract]) | 2551 |
| 1 | “Extracorporeal Shockwave Therapy”[Mesh] | 1172 |
