# Supplementary material for: Effectiveness of extracorporeal shock wave therapy for temporomandibular disorders: a systematic review and meta-analysis
Source: J Oral Facial Pain Headache. 2026 May 12;40(3):65–75. doi: 10.22514/jofph.2026.036 (PMC13223908; doi:10.22514/jofph.2026.036)
Supplement: Supplementary file 3 [file Supplementary-material-3.docx]

Supplementary material 3


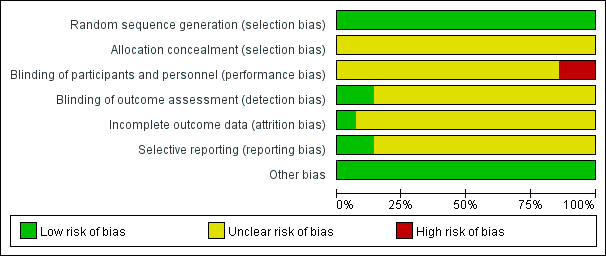


Supplementary Fig. 1. Risk of bias graph Review authors’ judgments about each risk of bias item for each included study.

Supplementary Table 1. GRADE Evidence Profile for ESWT as Adjunctive Therapy.

| Quality assessment | | | | | | | | No of patient | | | |
| --- | --- | --- | --- | --- | --- | --- | --- | --- | --- | --- | --- |
|  | No of studies | Design | Risk of bias | Inconsistency | Indirectness | Impression | Other consideration | ESWT + control | Control | Effect (95% CI) | Quality |
| VAS | 12 | RCTs | Very serious^1^ | No serious inconsistency | No serious indirectness | No serious impression | None | 468 | 479 | 0.94 (0.61~1.26) | Low |
| MMO | 10 | RCTs | Very serious^1^ | Serious^2^ | No serious indirectness | No serious impression | None | 409 | 420 | 0.69 (0.48~0.90) | Very Low |

^1^: high risk of bias; ^2^: moderate heterogeneity. VAS: visual analog scale; MMO: maximum mouth opening; RCT: randomized clinical trials; ESWT: Extracorporeal Shock Wave Therapy; CI: confidence interval.

Supplementary Table 2. GRADE Evidence Profile for ESWT as Monotherapy.

| Quality assessment | | | | | | | | No of patient | | | |
| --- | --- | --- | --- | --- | --- | --- | --- | --- | --- | --- | --- |
|  | No of studies | Design | Risk of bias | Inconsistency | Indirectness | Impression | Other consideration | ESWT | Control | Effect (95% CI) | Quality |
| VAS | 2 | RCTs | Very serious^3^ | Serious^4^ | No serious indirectness | No serious impression | None | 80 | 80 | −0.81 (−1.77~0.16) | Very Low |
| MMO | 2 | RCTs | Very serious^3^ | Serious^4^ | No serious indirectness | No serious impression | None | 80 | 80 | −1.84 (−18.43~14.74) | Very Low |

^3^: high risk of bias; ^4^: high heterogeneity. VAS: visual analog scale; MMO: maximum mouth opening; RCT: randomized clinical trials; ESWT: Extracorporeal Shock Wave Therapy; CI: confidence interval.


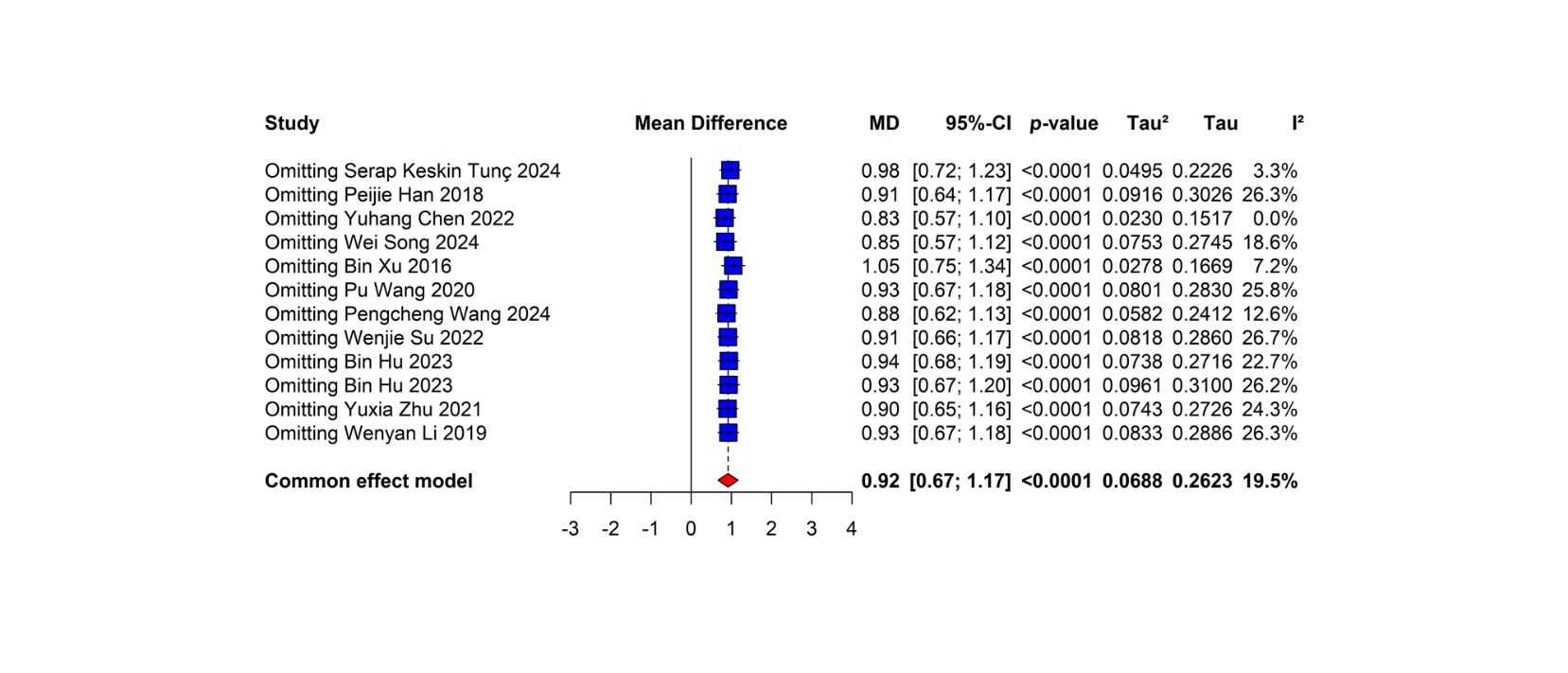


Supplementary Fig. 2. Leave-one-out sensitivity analysis forest plot of ESWT in Conjunction with Other Treatments for pain. MD: mean difference; CI: confidence interval.


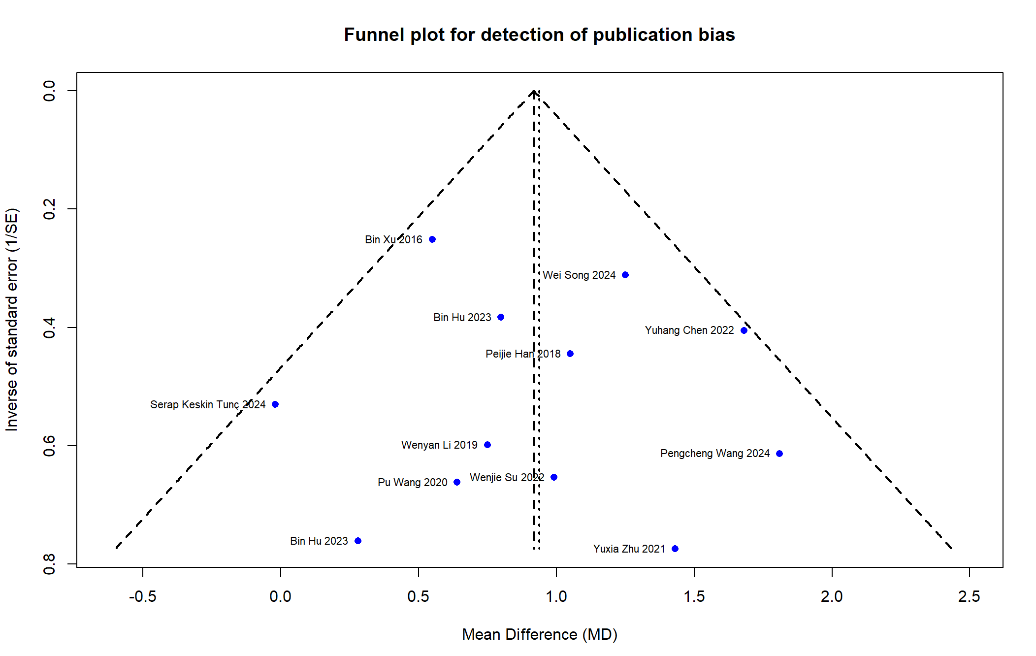


Supplementary Fig. 3. Publication bias heterogeneity funnel plot for pain (ESWT in Conjunction with Other Treatments). A funnel plot was used to assess the risk of publication bias. The diagonal lines represent the 95% confidence limits. SE: standard error; MD: mean difference. A random-effects model was used.


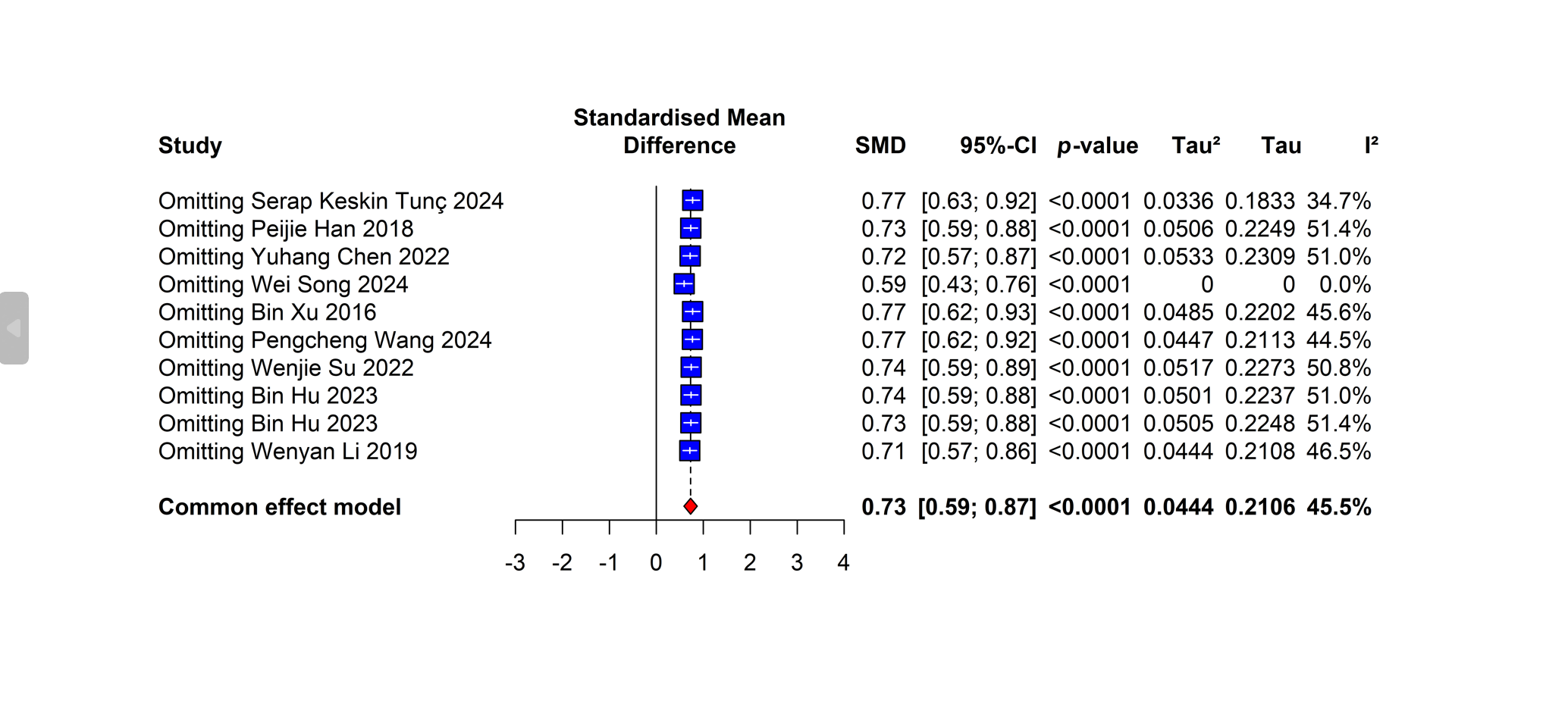


Supplementary Fig. 4. Leave-one-out sensitivity analysis forest plot of ESWT Conjunction with Other Treatments for maximal mouth opening. SMD: standardized mean difference; CI: confidence interval.


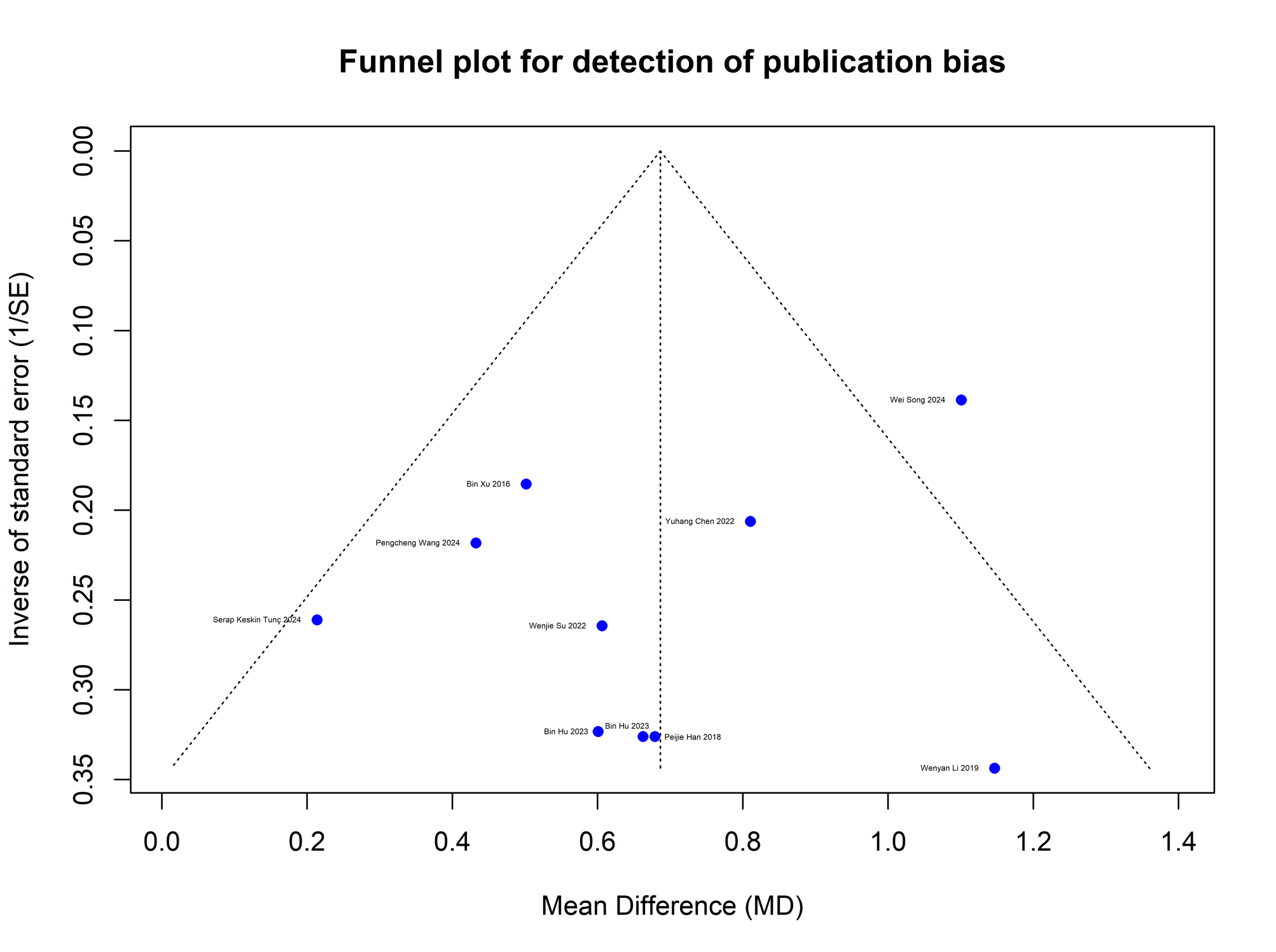


Supplementary Fig. 5. Publication bias heterogeneity funnel plot for maximal mouth opening (ESWT in Conjunction with Other Treatments). A funnel plot was used to assess the risk of publication bias. The diagonal lines represent the 95% confidence limits. SE: standard error; MD: mean difference. A random-effects model was used.
